# Silence in the EHR: infrequent documentation of aphonia in the electronic health record

**DOI:** 10.1186/1472-6963-14-425

**Published:** 2014-09-23

**Authors:** Megan A Morris, Abel N Kho

**Affiliations:** Mayo Clinic Department of Health Science Research, 200 1st Street SW, Rochester, MN 55905 USA; Department of General Internal Medicine and Geriatrics, Northwestern University, Chicago, IL USA

**Keywords:** Communication disabilities, Electronic Health Records, Patient-provider communication

## Abstract

**Background:**

To begin to deliver patient-centered care, providers need to be aware of when a patient has a communication disability and what communication methods to use with the patient. The aim of the study was to describe if and how patients’ communication disabilities are documented within electronic health records (EHR).

**Methods:**

A retrospective manual chart review of all inpatient and outpatient clinical encounter notes within the EHR for patients who had undergone a laryngectomy at Northwestern Memorial Hospital (Chicago, IL) between 2000–2013. We selected patients who had undergone a laryngectomy as the patient population as we were able to easily identify the patients through Common Procedural Terminology (CPT) codes.

**Results:**

We identified 81 patient charts with 7484 encounter notes. Of the 81 patient charts, 58 (72%) had at least one encounter note with a communication notation. Excluding speech-language pathology notes, 1164 (16%) of all encounter notes included some notation of the patients’ communication abilities. We coded the communication notations into four categories. 1) Descriptions of communication abilities appeared in 663 (9%) of all encounter notes, 2) descriptions of communication methods appeared in 590 (8%) of all encounter notes, and the last two categories 3) medical management and 4) referrals to speech-language pathology services each appeared in 148 (2%) of all encounter notes. While all patients had the same type of communication disability, aphonia, providers used 39 different terms and phrases to describe aphonia.

**Conclusions:**

Patients’ communication abilities were infrequently documented in the EHR. When providers did document a patient’s communication disability or method, they used inconsistent descriptions, suggesting a lack of standardized language. Further work is needed to determine how to consistently and accurately document patients’ communication abilities so staff and providers can quickly recognize how best to communicate with patients with communication disabilities.

## Background

Patients with communication disabilities including speech, language, and hearing disabilities can present with significant challenges for communication in the clinical environment, leading to potentially poorer quality of care [1–5]. In previous studies, patients reported benefiting from health care providers and staff members using communication strategies, such as gesturing, allowing the patient extra time to communicate, and maintaining eye contact while speaking [4, 6–8]. In order for providers and staff to use such strategies, they first need to be aware of a patient’s communication disability, as well as accommodations the patient requires. Documentation of communication abilities and accommodations in the electronic health record (EHR) potentially provides an effective and efficient method for this information to be documented and shared across providers and staff.

In 2010 the Joint Commission released a report entitled "Patient and Family Centered Care: A Roadmap for Hospitals", in which they recommend regular documentation of patients’ communication abilities in the medical record [9]. The stated purpose is to inform providers of when a patient has a communication disability and provide guidance on how best to communicate with the patient. The report does not outline how providers should document patients’ communication abilities, including what information to provide [10]. To date, only two studies have examined the documentation of communication abilities in the EHR. While providing important preliminary data, these studies focused on select environments, the intensive care unit (ICU) and outpatient physician visits, and gave limited information on the type of language providers used in the documentation [11, 12].

Identification of patients with communication disabilities within the EHR is challenging. First, while International Classification of Diseases-9 (ICD-9) codes for communication disabilities exist, communication disabilities are typically the result of another condition (e.g.: a patient is dysarthric as a result of a stroke). Providers often document the main diagnosis ICD-9 code, not the communication disability code. Second, communication disabilities can decrease in severity, as in the case of recovery from a stroke. A lack of communication disorder ICD-9 code or documentation of communication abilities could either be a result of the patient no longer having a communication disability or the provider not documenting the information.

In the current study we set out to identify the comprehensiveness of documentation of communication abilities within the EHR at a large academic medical center. We hypothesized that identifying patients with communication disabilities through ICD-9 codes or key communication disability terms would be inadequate, and so conducted an in-depth manual chart review of a select patient population.

## Methods

### Ethics statement

Study procedures were reviewed by the Northwestern University Human Subjects Board and determined exempt as we did not abstract any identifiable information from the EHR.

### Study population

We selected patients who had undergone a laryngectomy at Northwestern Memorial Hospital between January 2000 and July 2013. Patients with laryngectomies uniformly have the same type and severity of communication disability. While prosthetic and alternative means of communicating exist, all patients will have persistent communication disabilities. We were able to identify the patients within the EHR through laryngectomy procedure codes, which additionally provided an exact date in which the communication disability began.

### Data extraction

We queried the Northwestern Enterprise Data Warehouse to retrieve all patient charts that contained a laryngectomy Common Procedural Terminology (CPT) code within our timeframe. We identified 92 patients (see Table 1 for the list of CPT codes and frequencies) from a population of 1,525,336 distinct patients. Through a manual chart review, we determined that 10 patients did not have a laryngectomy and one patient had no electronic health records, thus we excluded these patients. The remaining 81 patient charts had one or more of the following CPT codes: 31360, 31365, 31367, 31390, and 31395. Collectively, these five CPT codes yielded three false positives (the patients did not undergo a laryngectomy) and had a positive predictive value of 0.96, or were able to correctly identify patients with laryngectomies in 96% of cases.

**Table 1:** **CPT codes used in the data extraction**

Procedures	CPT code	Number of patient charts	Patient did not have a laryngectomy
Laryngectomy, total, without neck dissection	31360	65	2
Laryngectomy, total, with neck dissection	31365	10*	0
Laryngectomy, supraglottic without neck dissection	31367	3*	1
Laryngectomy, supraglottic with neck dissection	31368	0	0
Pharyngolaryngectomy, with neck, without reconstruction	31390	7	0
Pharyngolaryngectomy, with neck, with reconstruction	31395	2**	0

*1 patient chart additionally had the 31360 CPT code.

**1 patient chart additionally had the 31390 CPT code.

We began by calculating the frequency of documentation of communication disorder ICD-9 codes across the 81 patient chart notes. We searched for all communication disorder ICD-9 codes, which all begin with the following numbers: 438.1, 784.3 and 784.4. Next, we conducted a manual chart review of all outpatient and inpatient clinical encounter notes following the patients’ laryngectomy procedure. Encounter notes included in the analysis were notes in which a provider interacted with the patient or a patient surrogate. We excluded informational notes including lab reports, radiology imaging reports, procedure reports, patient instructions, advance directives, and fall risk notes. For the encounter notes that met our criteria, we recorded any documentation that provided insight into the patient’s communication abilities, including the exact language used and where in the encounter note the documentation appeared. Two members of the research team independently coded 15% of all patient charts. Cohen’s Kappa ranged from 0.85-0.94 and percent agreement ranged from 97-100%.

We entered all the notes into NVivo 10.1 (QSR International Pty Ltd.) and conducted a content analysis, or an analysis of the text used in the communication ability documentation. We were interested in exploring what language providers used to describe communication disabilities and in what context did the notation occur [13]. For this we excluded all speech-language pathology notes as the purpose of the majority of these notes were to describe therapy to address and mitigate the communication disability, and therefore would skew our results. We categorized the communication ability documentation notes into four categories: (1) description of the patient’s communication abilities, (2) description of the communication methods the patient used or that the provider recommended the patient to use, (3) referral to speech-language pathology services, and (4) discussion of medical management of the patient’s speech abilities.

## Results

The 81 identified patient charts yielded 7,484 encounter notes (2,028 outpatient and 5,456 inpatient notes) that met our inclusion criteria. Patients’ mean age at the date of the laryngectomy was 64 years (range: 45–82 years); 75% of patients were male and 72% were white.

### ICD-9 codes

Providers used four communication ICD-9 codes; 784.3 "Aphasia" (one of the patients had a stroke following his laryngectomy), 784.41 "Aphonia", 784.49 "Other voice and resonance disorders", and 784.5 "Other speech disturbance" (see Table 2). A communication ICD-9 code was present in at least one encounter note in 29 or 36% of all 81 patient charts.

**Table 2:** **Communication ICD-9 codes**

ICD-9 code	Frequency across all encounter notes (n = 7,484)	Frequency across patient charts (n = 81)
784.3 Aphasia	1	1
784.41 Aphonia	340	27
784.49 Other voice and resonance disorders	2	2*
784.5 Other speech disturbance	6	2**
Total	349	29***

*Two charts also have 784.41 code.

**One chart also has 784.41 code.

***Excludes duplicate charts.

### Documentation of communication abilities

At least one encounter note with communication ability documentation appeared in 58 or 72% of the 81 charts, that is to say, 23 (28%) of charts had no provider notes with documentation of the patient’s communication ability. This number includes charts with speech-language pathology notes, although all charts with communication ability documentation from a speech-language pathologist also had communication ability documentation by another type of provider. Thirty one charts had at least one note with a description of the patient’s communication ability but no communication ICD-9 codes, 28 charts had at least one communication ICD-9 code and a communication ability note, and one chart had just a communication ICD-9 code but no communication ability notes. Finally, 22 or 27% of the 81 charts had neither a communication ICD-9 code nor a note with documentation of the patient’s communication abilities.

Analyzing the individual notes within the patients’ charts and excluding the speech-language pathology notes (n = 210), 1164 or 16% of all encounter notes included some documentation of the patient’s communication abilities (see Table 3). Of the notes that included documentation of communication abilities, 56% (n = 655) described the patient’s abilities, 52% (n = 605) described the method the patient used to communicate, 10% (n = 116) discussed medical management of the patient’s speech, and finally 10% (n = 116) were a referral to speech-language pathology services. Some statements were coded into more than one category. For example, "patient is nonverbal and uses pencil and paper to write" was coded as both a description and a method. Examining the frequency across all encounter notes, excluding speech-language pathology notes, (n = 7,374), 9% described the patient’s communication abilities and 8% described the method the patient used to communicate. Within structured notes, providers documented communication abilities within the following note subsections: subjective, history, objective, assessment, physical exam, summary and plan. No clear pattern emerged.

**Table 3:** **Communication ability notes**

Category	Outpatient notes (n = 340)	Inpatient notes (n = 840)	Example
Description	196 (58%*)	459 (55%*)	Patient unable to speak.
Method	169 (50%*)	436 (52%*)	Communicates by writing.
Medical management	67 (20%*)	49 (6%*)	We discussed the placement of tracheoesophageal puncture.
Referral	43 (13%*)	74 (9%*)	Speech pathology was consulted regarding speech rehabilitation.

*Percentage of communication ability notes.

### Language in the communication ability notes

All study patients were unable to produce a natural voice, rendering all aphonic. Providers used 39 different terms and phrases to describe aphonia. The most frequently used terms, which represented 55% of the descriptions, included: nonverbal, unable to or cannot speak, and aphonic or aphonia. In 14% of the descriptions, providers described the patients’ communication as normal or non-disordered. Patients used a variety of methods and strategies to communicate, including: writing, gesturing, having a family member speak for the patient, mouthing words, and using a tracheoesophageal puncture or an electrolarynx. The electrolarynx was a commonly cited strategy, with providers mentioning it in 44% of patient charts in which there was a communication ability notation. Providers used 35 different terms to describe the device. Example terms included: artificial larynx, electronic vocalization, laryngeal voice machine, and voice box adapter.

## Discussion

Documentation of patients’ communication abilities in the EHR would assist in alerting providers and staff of when a patient has communication difficulties and how to best communicate with the patient. Despite a policy recommendation for this documentation, we found that for patients who had undergone a laryngectomy, a population in which all have a communication disability, providers rarely described patients’ current communication abilities or methods used to communicate. Additionally, providers rarely used communication disability ICD-9 codes. Over a quarter of the patients had absolutely no documentation of their communication disability, rendering it impossible to determine from their chart how the patient was communicating. When providers did describe the patients’ communication abilities, they used inconsistent terms that potentially provided limited information for how to communicate with the patient. For example, a patient could be "nonverbal" for a variety of reasons, including a severe language disability such as aphasia or congenital deafness, conditions which require quite different accommodations. Finally, no uniformity existed for where communication ability information appeared within the note. Consequently, quickly identifying patients with communication disabilities and the methods they use to communicate would be challenging.

Several previous studies have explored documentation of communication disabilities in the EHR [11, 12]. The first study found that in patients with moderate to severe hearing loss, physicians documented hearing loss in 28% of outpatient notes [11]. The study authors noted that information about hearing abilities frequently appeared in the physical exam section and providers often used the "HEENT" or "head, eyes, ears, neck and throat" review of systems acronym. This structured format gave the providers a framework for reporting hearing disabilities. In a study of ventilated, non-verbal patients in the ICU, researchers found that 72% of the patient charts had at least one documentation of a patient’s communication [12]. We found a similar percentage (72%) of patients’ charts with at least one note with documentation of communication abilities suggesting that our findings might apply to different types of communication disabilities and healthcare environments.

Several possible factors could have contributed to our findings of a low rate of documentation of communication abilities. We did not record the rate of documentation per provider. It is possible that once a provider was aware of and documented a patient’s communication disability, he/she did not think it necessary to document the disability in future encounter notes. While this could account for the low rate of documentation, there were a large number of encounter notes and quite a low rate across the notes. Furthermore, providers should regularly document the methods patients use to communicate as this could change across encounters. Finally, some might argue that patients with an electrolarynx or a speech prosthesis, such as a tracheoesophageal puncture, are "cured" and therefore it is not necessary to document their communication disability. Despite augmentation, these patients still have speech disorders and the method they use to correct the speech should be documented so other providers and staff know how the patient communicates.

The results of this study are timely in light of recent EHR policy recommendations. The Health Information Technology for Economic and Clinical Health Act (HITECH) of 2009 outlines requirements for achieving meaningful use standards for EHRs. While Stages 1 and 2 of the meaningful use standards did not require documentation of communication disabilities, Stage 3 does. Specifically, draft standards recommend documenting all disabilities (which would include communication disabilities) on registration as part of demographics [14, 15]. More recently, the United States Department of Health and Human Services Department released proposed functionality recommendations for EHRs and suggested that healthcare organizations document patients’ disability status in the EHR and link the information to available disability accommodations [16].

Documenting communication abilities and accommodation needs in the demographic section of the EHR would (1) negate the need for documentation in each encounter note, (2) provide a consistent location, and (3) create a potentially effective and efficient method for dissemination to providers and staff.

## Conclusions

As stated in the HITECH Stage 3 draft standards, future research is needed to develop methods and standards for how to collect disability status. Standardized language to document all types of communication disabilities needs to be developed. Additionally, simple decision support could be built into the EHR to generate patient and family requests for disability accommodations. For example, if upon registration a patient is noted to have difficulty comprehending language due to a diagnosis of aphasia, the patient and his/her family could then be asked about specific strategies providers and staff should use with the patient to facilitate communication (e.g.: gestures or writing down key words). The language to describe communication disabilities and disability accommodations would need to be concise yet informative so providers and staff at all levels know how best to communicate with each patient. Consistent and accurate documentation of communication disabilities that is available in the same location will assist staff and providers in quickly recognizing how best to communicate with patients with communication disabilities and potentially provide higher quality of care.

## Abbreviations

EHR: Electronic health records
ICD-9: International Classification of Diseases
CPT: Common Procedural Terminology.
